# Growth performance, meat quality, strength of jejunum and leg bones of both sexes Cherry Valley ducks fed with zeolite

**DOI:** 10.1038/s41598-024-54393-2

**Published:** 2024-02-16

**Authors:** Sebastian Wlaźlak, Jakub Biesek, Mirosław Banaszak

**Affiliations:** Department of Animal Breeding and Nutrition, Faculty of Animal Breeding and Biology, Bydgoszcz University of Science and Technology, 85-084 Bydgoszcz, Poland

**Keywords:** Zoology, Environmental sciences

## Abstract

Zeolite, as a natural mineral, could be a good additive for ducks, in line with pro-environmental trends. The study aimed to evaluate zeolite additives in feed for broiler ducks of both sexes on production results, meat quality, and the strength of the jejunum, tibia, and femur. The experiment used 200 Cherry Valley ducks, divided into a control group of males (CM) and females (CF) and an experimental group of males (ZM) and females (ZF). In the control groups, a commercial diet was used. In the experimental groups, 1% zeolite was added. The ZM group demonstrated higher body weight and weight gain than the CM group. Zeolite reduced the feed conversion ratio. A higher liver weight was found in the experimental group (ZM). Notably, zeolite influenced the weight of male pectoral muscles. Higher water loss in the pectoral muscles and higher protein content in the leg muscles were found in the same group. Females had a higher weight of neck and wings with skin. Female pectoral muscles had lower protein and water content. Zeolite in feed at a 1% level for broiler ducks could be recommended as a natural additive that positively affects the ducks' production results concerning good quality meat.

## Introduction

The production of broiler ducks in Poland in 2022 amounted to 67.8 thousand tons of carcass weight. It indicates a stable third position in the European Union for several years^1^. Duck meat is a product with high nutritional value, high content of fatty acids, and higher fat content than chicken meat (higher palatability). It is gaining popularity among consumers and may be competitive with meat from other poultry species^2,3^.

Feed additives have been used in poultry production for many years, which can result in better utilization of nutrients from feed, higher body weight gain, and lower feed consumption, as well as modulate the mechanisms of the immune system and optimize production efficiency. These include probiotics, prebiotics, synbiotics, eubiotics, phytobiotics, herbs, organic acids, essential oils, and minerals^4–7^. The use of substances with health-promoting properties is part of the European Green Deal strategy due to the potential reduction of antibiotics in animal production^8^.

Zeolites are a group of natural minerals belonging to aluminosilicates (analcime, chabazite, laumontite, and mordenite). These clay minerals with a porous structure have some properties that allow them to be used in many industrial sectors (agriculture, horticulture, household products)^9^. In animal production, aluminosilicates are used as feed additives as well as bedding additives^10–12^, primarily due to their high ability to absorb water and also harmful gases (especially ammonia) and mycotoxins, as well as reducing the population of harmful insects^9,13,14^.

Except for the positive impact of these substances on the environmental conditions in the livestock houses, the beneficial effect of aluminosilicates on selected production parameters of broiler chickens (higher body weight gain, lower mortality)^15^, and histomorphometry parameters of the jejunum, as the elongation of intestinal villi^16^ was notably found. In the study by Hcini et al.^17^, 1 and 2% zeolite were added to feed for turkeys. It was found that the experimental groups were characterized by higher growth efficiency and higher meat quality (including the content of polyunsaturated fatty acids). Feeding geese with the addition of zeolite, including nanostructured zeolite, may increase the body weight of birds and modify the content of selected chemical traits of meat^18^.

The beneficial effect of aluminosilicates on the body of birds may occur in the case of a protective effect on the liver and activation of metabolic processes or antitoxic effects^19^. Changes in the characteristics of blood serum, the weight of selected organs, and the chemical composition of muscles were also observed^20^. Many studies have found changes in selected carcass characteristics and physicochemical indicators of poultry meat^11,21–24^.

Research on the use of aluminosilicates in feeding broiler ducks is largely limited. In a pilot study, Biesek et al.^25^ showed that zeolite at a level supplementation of 4% in the feed influenced negative on body weight gain and water-holding capacity (7 weeks of rearing). It was also noticed that the yellowness was higher in leg muscles of ducks fed with zeolite. Simultaneously, it was concluded that too high an additive level in the feed may be a limiting factor. These results were inconclusive and required further experiments to verify the effect of zeolite thoroughly on the production results and meat quality of broiler ducks.

The study aimed to evaluate the use of zeolite addition at the level of 1% to the commercial diet of Cherry Valley broiler ducks and drakes on production results, carcass composition, meat quality, and strength of the jejunum, femur, and tibia.

## Results

### Growth performance

Significantly higher body weight of drakes was found in the zeolite group compared to the control group on days 28 (P = 0.048) and 42 (P = 0.041). Drakes fed with a diet supplemented with zeolite also achieved significantly higher (total) body weight gains (P = 0.040) and average daily body weight gains (P = 0.040). A significant effect of zeolite on the reduction of FCR was found when ducks were fed grower feed (P = 0.034) (Table 1).

**Table 1:** Growth performance of broiler ducks as influenced by zeolite, sex and its interaction.

Item^c^ n = 10	Group^d^				SEM	P value		
	CM	CF	ZM	ZF		Zeolite × Sex	Zeolite	Sex
Viability (%)	96.00	92.00	94.00	94.00	1.338	0.490	1.000	0.470
BW (g)								
Day 1	61.20	60.88	60.76	60.92	0.183	0.549	0.599	0.834
Day 28	1838.42^b^	1884.77^ab^	1912.19^a^	1854.30^ab^	12.911	0.048	0.416	0.830
Day 42	2883.29^b^	3007.22^ab^	3130.84^a^	2997.87^ab^	33.264	0.041	0.072	0.948
BWG (g)								
Days 1–28	1777.22	1823.89	1851.43	1793.38	12.871	0.046	0.411	0.832
Days 29–42	1044.87	1122.45	1218.65	1143.57	25.570	0.119	0.054	0.981
Total	2822.09^b^	2946.34^ab^	3070.08^a^	2936.95^ab^	33.265	0.040	0.072	0.949
ADBWG (g)								
Days 1–28	63.47	65.14	66.12	64.05	0.460	0.046	0.411	0.832
Days 29–42	80.37	86.34	93.74	87.97	1.967	0.119	0.054	0.981
Total	67.19^b^	70.15^ab^	73.10^a^	69.93^ab^	0.792	0.040	0.072	0.949
FI (g)								
Days 1–28	3104.60	3180.98	3251.99	3256.21	35.119	0.613	0.115	0.580
Days 29–42	4378.92	4589.14	4451.14	4470.40	65.222	0.496	0.864	0.394
Total	7621.34	7994.40	7771.89	7795.68	113.059	0.472	0.919	0.395
ADFI (g)								
Days 1–28	199.59	227.21	232.29	232.59	6.401	0.278	0.141	0.287
Days 29–42	235.85	218.53	211.96	212.88	7.931	0.590	0.366	0.618
Total	181.46	190.34	185.04	185.61	2.692	0.472	0.919	0.395
FCR (kg/kg)								
Days 1–28	1.75	1.74	1.76	1.82	0.014	0.252	0.163	0.337
Days 29–42	4.23	4.10	3.66	3.93	0.089	0.222	0.034	0.708
Total	2.70	2.71	2.53	2.66	0.034	0.378	0.099	0.338
EPEF	244.53	242.87	277.50	254.85	6.602	0.417	0.089	0.371
EBI	239.34	237.93	272.12	249.68	6.490	0.408	0.086	0.373

^a,b^Mean values with various letters in the row differ statistically significantly between all groups, including interaction (P < 0.05).

^c^*BW* body weight, *BWG* body weight gain, *ADBWG* average daily body weight gain, *FI* feed intake, *ADFI* average daily feed intake, *FCR* feed conversion ratio, *EPEF* European Production Efficiency Factor, *EBI* European Broiler Index.

^d^*CM* male control group, *CF* female control group, *ZM* experimental male group fed with zeolite, *ZF* experimental female group fed with zeolite.

### Carcass features

Slaughter yield was similar in all groups and ranged from 65.04 to 67.27 g/100 g of pre-slaughter body weight. Significantly higher liver weight was found in ducks from the ZM group compared to ZF and CF (P < 0.001). Males had significantly higher liver’ weight compared to females (P < 0.001). Ducks from the CM group had the significantly highest weight of the gizzard (P = 0.001). Significantly lower weight of the neck was demonstrated in the ZM group compared to CM and ZF (P < 0.001). The weight of pectoral muscles was significantly the highest in group ZM (P = 0.045). Significantly lower skin with subcutaneous fat weight was demonstrated in the CM group compared to ZM (P = 0.039) (Table 2).

**Table 2:** The carcass composition of broiler ducks as influenced by zeolite, sex and its interaction.

Item n = 10	Group^d^				SEM	P value		
	CM	CF	ZM	ZF		Zeolite × sex	Zeolite	Sex
g/100 g of pre-slaughter body weight								
Slaughter yield	65.71	67.29	66.14	65.04	0.339	0.116	0.183	0.730
Slaughter yield with offal	71.92	72.60	71.90	70.88	0.320	0.303	0.175	0.797
g/100 g of carcass with offal								
Heart	0.67	0.66	0.68	0.69	0.013	0.801	0.375	0.966
Liver	3.55^ab^	2.93^c^	3.80^a^	3.27^b^	0.083	< 0.001	0.076	< 0.001
Gizzard	4.42^a^	3.74^bc^	3.54^c^	4.28^ab^	0.100	0.001	0.404	0.874
g/100 g of carcass								
Neck	8.17^ab^	7.49^bc^	6.86^c^	8.75^a^	0.174	< 0.001	0.940	0.081
Pectoral muscle	15.96^bc^	18.19^ab^	18.20^a^	15.80^c^	0.417	0.045	0.929	0.915
Leg muscle	13.88	13.18	12.72	14.42	0.279	0.144	0.940	0.382
Skin with subcutaneous fat	18.51^b^	20.89^ab^	21.81^a^	20.95^ab^	0.433	0.039	0.051	0.389
Abdominal fat	0.64	0.69	0.59	0.70	0.046	0.852	0.847	0.412
Wings with skin	11.48	11.77	11.35	12.41	0.236	0.403	0.601	0.155
Carcass remains	31.35	27.80	28.47	26.98	0.614	0.376	0.117	0.036

^a,b,c^Mean values with various letters in the row differ statistically significantly between all groups, including interaction (P < 0.05).

^d^*CM* male control group, *CF* female control group, *ZM* experimental male group fed with zeolite, *ZF* experimental female group fed with zeolite.

### Meat quality traits of pectoral and leg muscles

The significantly highest pH was found in the meat of ducks from the CF group (P = 0.029, P = 0.014). This group also showed the lowest muscle lightness compared to CM and ZF (P = 0.003). The pectoral muscles from the ZM group were characterized by significantly higher WHC (P = 0.024; P = 0.004), protein content (P = 0.032; P = 0.014), and water content (P < 0.001). Significantly, the lowest salt content was found in the muscles of drakes of both groups compared to females (P < 0.001), as well as IMF compared to ZF (P = 0.002; P = 0.001). The leg muscles of ducks from CF had significantly the lowest WHC (P = 0.003; P = 0.011), protein content (P < 0.001), and water content (P < 0.001). The highest salt content was found in the muscles from the ZM group (P = 0.002), and the IMF content was found in the muscles of ducks from the ZF group (P < 0.001; P = 0.001) (Table 3).

**Table 3:** Meat quality traits of pectoral and leg muscles as influenced by zeolite, sex and its interaction.

Item^d^ n = 10	Group^e^				SEM	P value		
	CM	CF	ZM	ZF		Zeolite × Sex	Zeolite	Sex
Pectoral muscle								
pH_24 h_	5.87^ab^	5.95^a^	5.84^ab^	5.83^b^	0.017	0.029	0.014	0.289
L*	41.42^a^	36.67^b^	37.83^ab^	40.52^a^	0.551	0.003	0.908	0.356
a*	15.19	16.47	16.37	15.34	0.304	0.308	0.960	0.839
b*	3.47	2.12	2.73	2.79	0.240	0.270	0.936	0.181
Drip loss (%)	1.82	1.53	1.64	2.08	0.131	0.485	0.492	0.773
WHC (%)	32.68^ab^	31.29^b^	35.97^a^	34.94^ab^	0.618	0.024	0.004	0.332
Protein (%)	20.89^ab^	20.82^ab^	20.99^a^	20.67^b^	0.041	0.032	0.745	0.014
Collagen (%)	1.39	1.37	1.42	1.38	0.026	0.902	0.680	0.548
Salt (%)	0.26^b^	0.37^a^	0.29^b^	0.38^a^	0.012	< 0.001	0.451	< 0.001
IMF (%)	2.46^b^	2.80^ab^	2.51^b^	3.07^a^	0.069	0.002	0.249	0.001
Water (%)	77.05^a^	76.33^b^	76.91^a^	76.09^b^	0.089	< 0.001	0.286	< 0.001
Leg muscle								
L*	37.84	37.69	38.92	38.27	0.551	0.871	0.460	0.722
a*	15.49	14.37	14.56	14.18	0.351	0.579	0.432	0.294
b*	2.98	2.81	2.87	3.63	0.291	0.753	0.552	0.614
WHC (%)	25.65^b^	31.19^a^	29.76^a^	30.17^a^	0.598	0.003	0.200	0.011
Protein (%)	18.78^c^	19.26^b^	19.82^a^	19.12^b^	0.067	< 0.001	< 0.001	0.401
Collagen (%)	1.49	1.46	1.51	1.63	0.025	0.080	0.059	0.364
Salt (%)	0.54^ab^	0.52^b^	0.57^a^	0.51^b^	0.007	0.002	0.319	0.002
IMF (%)	5.80^b^	5.90^b^	5.48^b^	6.84^a^	0.113	< 0.001	0.173	0.001
Water (%)	74.42^a^	73.54^b^	73.64^b^	72.48^c^	0.126	< 0.001	< 0.001	< 0.001

^a,b,c^Mean values with various letters in the row differ statistically significantly between all groups, including interaction (P < 0.05).

^d^L* lightness, a* redness, b* yellowness, *WHC* water holding capacity, *IMF* intramuscular fat.

^e^*CM* male control group, *CF* female control group, *ZM* experimental male group fed with zeolite, *ZF* experimental female group fed with zeolite.

### Jejunum tensile strength and bones breaking strength

Significantly, the highest force needed for breaking the tibia was observed in the CM (292.30) and ZF (287.20) compared to the ZM (237.37) (P = 0.048). For other parameters, such as jejunum strength or femur strength, no statistically significant differences were found between the groups (P > 0.05) (Table 4).

**Table 4:** Jejunum tensile strength and breaking strength of tibia and femur bones as influenced by zeolite, sex and its interaction.

Item n = 10	Group^c^				SEM	P value		
	CM	CF	ZM	ZF		Zeolite × Sex	Zeolite	Sex
Jejunum								
Tensile strength (N)	8.85	9.61	8.38	9.94	0.823	0.919	0.965	0.495
Femur bone								
Breaking strength (N)	285.11	280.98	245.68	291.06	11.043	0.478	0.513	0.357
Breaking strength (N/g)	25.14	29.12	24.71	26.00	0.990	0.399	0.376	0.187
Tibia bone								
Breaking strength (N)	292.30^a^	270.52^ab^	237.37^b^	287.20^a^	7.781	0.048	0.224	0.374
Breaking strength (N/g)	18.43	19.13	15.62	17.04	0.791	0.420	0.123	0.512

^a,b^Mean values with various letters in the row differ statistically significantly between all groups, including interaction (P < 0.05).

^c^*CM* male control group, *CF* female control group, *ZM* experimental male group fed with zeolite, *ZF* experimental female group fed with zeolite.

## Discussion

In our research, the BW and ADBWG of ducks in the ZM group on days 28 and 42 were significantly higher compared to the CM group. In the experimental group, ducks had lower FCR than the control group. The positive effect of natural minerals in poultry rearing has also been found in previously conducted studies. Banaszak et al.^21^ point out that broiler chickens' significantly highest BW and BWG were observed in the group fed with a mixture of halloysite and zeolite and added to the litter. According to Karamanlis et al.^26^, zeolite in feed (2%) and litter (2 kg/m^2^) also increased the BW of birds compared to the control group. Contrary to our research, zeolite at the level of 4% in the feed reduced BWG (by 0.34 kg) and increased FCR (by 0.50 kg/kg) in commercial ducks during 49 days of rearing^25^. An et al.^27^ have shown that feeding with 1% zeolite improved production results of Arbor Acres chickens' production results at raised temperatures. Zeolite may have protective properties against the harmful effects of heat stress in birds^27^.

Selected aluminosilicates may benefit the selected morphometric features of the jejunum. Halloysites in feed and litter increased the height and area of intestinal villi of broiler chickens^16^. Corresponding results were shown by supplementation with 3% zeolite in feed. Moreover, a higher thickness of the mucosa of the jejunum and ileum was noted^28^. The jejunum is the middle of the small intestine^29^, where digestion and intensive absorption of nutrients such as sugars, fats, amino acids, and minerals occur^30^. Zeolite can stimulate the secretion of digestive enzymes (amylase, lipase, and jejunal trypsin) and increase the digestibility of crude protein^31^. This ability may explain the improved BWG of broiler chickens. Similar conclusions were presented by Tang et al.^32^, in the study concerning the chickens feeding with zeolite containing zinc or Aigamo ducks fed with a mixture of zeolite, plant extract, and vermiculite^33^. The beneficial effect of zeolite on the digestion and absorption process has been demonstrated in other species of livestock, such as pigs^34,35^ and cows^36^. Due to the above properties, this could result in better production rates of ducks from the ZM group. In addition to the parameters determining the absorption of nutrients in the intestines, this mineral may be an immunomodulating factor of the immune system (expression of interleukin and interferon genes) and improve intestinal tightness^37^. It constitutes another health-promoting property, which could indirectly translate into better production results.

Zeolite significantly affected the weight of the liver and gizzard, unlike Pavlak et al.^38^, which did not indicate significantly different liver weights after using different doses of zeolite in broiler chickens' feed (0.25%, 0.5%, and 1%). Cited authors only indicated affection on the pancreas weight. Changes in the weight of the pancreas could result from the intensity of enzyme secretion. This relationship could indicate the highest liver weight in drakes fed with zeolite. Liver cells are the target site for neutralizing toxic substances absorbed in the gastrointestinal tract^39^. Zeolite is characterized by a porous structure that facilitates the absorption of toxic substances, e.g., mycotoxins^40^. A few studies have shown reduced liver weight by deoxynivalenol or zearalenone in chickens^41,42^. It constitutes a specific protective barrier for the liver and the entire body.

The significantly different weight of chicken gizzard depending on the method of zeolite application was confirmed by Basha et al.^43^. However, Banaszak et al.^11^ and Abdelrahman et al.^24^, did not noted any affect on the gizzard weight. Sand grains and natural minerals (grit) particles can affect digestion and gizzard weight^44^. In the presented research, the mineral was used in dusty form. Scientific research in this area is focused primarily on *Gallinaceous* poultry. Significantly, the neck's highest weight were found in the ZF group. Drakes from the ZM group were characterized by a significantly higher pectoral muscle weight than CM group (over 60.00 g).

The lowest weight of skin with subcutaneous fat occurred in the CM group. Contrary to our results, different doses of zeolite in the feed did not affect the weight of chicken pectoral muscles^45^. Similar results were shown by Saçakli et al.^46^ using only clinoptilolite or in combination with phytase. The pectoral muscle weight is a crucial element of the poultry carcass with high economic importance^47^. The increase in muscle weight depends on the rate of increase in the length and diameter of specific muscle fibers (the number of fibers is constant), which is determined, for example, by the diet^48^. The higher pectoral muscle weight could be influenced by better nutrient digestibility after using zeolite. Reports by other authors are inconsistent, as the protein content in the feed determines the highest performance of pectoral muscles, as opposed to digestibility^49^. Drakes were characterized by a higher weight of the liver neck but lower wing weight than females. Kaewtapee et al.^50^ found that Cherry Valley drakes had significantly higher weights of the gizzard and pectoral muscles. The influence of sex on carcass characteristics may be determined by differences in physiology (hormone secretion and metabolic processes).

The pH of the pectoral muscles of all groups was in the range of 5.83–5.95. It corresponds to the results of poultry meat pH from the other authors^12,25^. Pectoral muscles from the CM and ZF groups were characterized by significantly higher lightness. Lower WHC occurred in the pectoral muscles of the control group. The appropriate pH value of meat 24 h after slaughter indicates the course of the glycolysis process and the formation of H + ions during post-slaughter ripening^51^. It also affects the texture and color of the meat. Meat with a higher pH is characterized by a darker color^52^, which is confirmed by the results of meat from females from the control group (the highest pH value, the lowest L* value). Shabani et al.^53^ demonstrated the possibility of limiting lipid oxidation in the meat of slaughtered chickens through the action of aflatoxins after using zeolite (0.75 and 1%). This process affects changes in meat color—discoloration due to oxidation of heme dyes^54^, which could confirm a significantly higher L* value in the pectoral muscles of females of the experimental group compared to females of the control group. Similar to our research, Biesek et al.^25^ also found significantly higher water loss from ducks' pectoral and leg muscles at 6 weeks. WHC is an essential indicator of the technological suitability of meat for further processing and its sensory value^55^. It is related to the denaturation of muscle proteins during post-slaughter meat maturation (pH drop). Thus, more free water is created, increasing WHC^56^. The low pH of meat determines its lighter color, soft consistency and high water loss (low water absorption)—meat with a PSE defect (pH ≤ 5.8). Too high pH of the meat causes its darker color and slight drip loss (DFD meat; pH ≥ 6.3)^57,58^.

The chemical composition of the pectoral muscles indicates significant differences by sex (salt, IMF, and water content in the pectoral and leg muscles and protein content in the pectoral muscles). After zeolite application, drake pectoral muscles had the highest protein content, and female pectoral muscles had the highest salt and IMF content. A significantly higher protein content in leg muscles was found in the experimental groups. Safaei et al.^59^ revealed a reduction in the IMF content and no changes in the protein content in the meat of broiler chickens fed with aluminosilicates (zeolite, bentonite, kaolin). Other studies also indicated worse protein content in duck meat^25^. The chemical composition of meat largely depends on nutritional factors^60^ and affects its dietary value^61^ and sensory properties^62^.

The tibia of drakes fed with zeolite were characterized by lower fracture strength than the bones from the ZF and CM groups. Biesek et al.^25^ found no effect of zeolite addition on the strength parameters of tibia bones of ducks of various origins, or Eleroğlu et al.^63^ fed chickens with Ca–zeolite addition. According to Safaeikatouli et al.^64^, 3% bentonite or zeolite improved the properties of the chicken tibia (weight/length index, strength index), possibly due to higher calcium absorption.

To sum up, feeding broiler ducks with 1% zeolite addition positively affected selected production results, especially in the male group. In particular, lower FCR was found in the experimental group in both sexes. The critical aspect is in the features of pectoral muscles. Zeolite increased the WHC of pectoral muscles, and in leg muscles, the protein and IMF content improved, and the water content decreased. It is justified to recommend 1% zeolite in the feed for broiler ducks. It has been shown that the sex of ducks may differentiate the production results and carcass characteristics. When analyzing our results and considering the available literature, zeolite is crucial in the digestion of nutrients and the immunity of ducks; however, it should be additionally studied. Taking up the presented research issues allows for a new perception of natural additives such as zeolite in duck nutrition. Thus, its favorable properties can contribute to improving production efficiency indicators and obtaining good-quality meat.

## Material and methods

The consent for research was obtained from the Institutional Animal Care and Use Committee of the Bydgoszcz University of Science and Technology (Local Ethical Committee, No. 2/2022). The ethics committee of Bydgoszcz University of Science and Technology approved the experimental protocols presented in this study. The National Ethical Committee for Animal Experiments recommendations and the ARRIVE guidelines were also considered. The experiment followed Directive 2010/63/EU of the European Parliament and the Council. All methods used in the experiment were applied in accordance with applicable guidelines and requirements.

### Animals and diets

In the experiment, 100 females (ducks) and 100 males (drakes) of Pekin Cherry Valley SM3 Medium ducklings were used (Tulce, Greater Poland Voivodeship, Poland). Birds were divided into 4 groups of equal number, 5 repetitions (pens) each. There was a control group of males (CM), a control group of females (CF), and an experimental group of males (ZM) and females (ZF).

Rearing lasted 42 days. The environmental conditions in the broiler duck house were adapted to the recommendations for keeping broiler ducks. On the first day of rearing, the temperature was 26 °C, which was gradually lowered to 18 °C (after 4th week of life). An additional heat source with a temperature of 30 °C (and lowered within the time) was placed in each pen (for 4 weeks). The relative humidity was in the range of 60–70% and the air movement was 0.2–0.3 m/s. The concentration of harmful gases was monitored to ensure that it did not exceed 3000 ppm—CO_2_, 20 ppm—NH_3_ and 5 ppm—H_2_S, respectively. The pen area was 2 m^2^ and the stocking density did not exceed 17 kg/m^2^. Chopped wheat straw was used as bedding. Feed and water were provided ad libitum. Rearing was divided into two feeding periods. From day 1 to 28, the ducks were fed a commercial diet of the starter type. From day 29 to 42, a grower-type commercial diet was used. The commercial diet was isocaloric and isoprotein. The composition of commercial diets corresponded to the nutritional recommendations for broiler ducks^65^. The control groups were fed commercial diets without additives throughout the rearing period. In the experimental groups, birds were fed a commercial diet supplemented with 1% zeolite, previously thoroughly mixed mechanically^66^. Chemical composition of zeolite: SiO_2_ (silicon dioxide)—71.30%; Al_2_O_3_ (aluminum oxide)—13.10%; CaO (calcium oxide)—5.20%; K_2_O (potassium oxide)—3.40%; Fe_2_O_3_ (iron (III) oxide)—1.90%; MgO (magnesium oxide)—1.20%; Na_2_O (sodium oxide)—1.30%; TiO_2_ (titanium oxide)—0.30%; Si/Al (silicon / aluminum)—5.40%; Clinoptilolite—84.00%; Cristobalit—8.00%; Mica clay—4.00%; Plagioclases—3.50%; Rutile—0.20%. Specific surface area of 30–60 m^2^/g, a bulk density of 1.60–1.80 kg/m^3^ and weight of 2.20–2.44 kg/m^3^.

### Diet composition

Starter feed contained: maize, wheat, soybean extraction meal, wheat bran, sunflower extraction meal, hulled sunflower seeds, barley, rapeseed extraction meal, wheat gluten feed, calcium carbonate, animal fat, monocalcium phosphate, vegetable oil and fat (raw sunflower), sodium chloride, and sodium sulfate, crude protein (19.5%), ether extract (3.9%), crude fiber (4.2%), lysine (0.93%), methionine (0.42%), threonine (0.72%), calcium (0.85%), phosphorus (0.69%), sodium (0.17%), vitamin A (10,000 IU), vitamin D3 (3000 IU), vitamin E (25 IU). Grower feed contained: maize, wheat, wheat bran, soybean extraction meal, sunflower extraction meal, from dehulled sunflower seeds, triticale, rapeseed extraction meal, animal fat, calcium carbonate, monocalcium phosphate, sodium chloride, calcium bicarbonate, crude protein (17.1%), ether extract (3.7%), crude fiber (4.5%), lysine (0.87%), methionine (0.37%), threonine (0.61%), calcium (0.81%), phosphorus (0.66%), sodium (0.16%), vitamin A (10,000 IU), vitamin D3 (3000 IU), vitamin E (25 IU) (De Heus, Łęczyca, Polska). Commercial diet from each feeding period and group was collected (approximately 500 g) into 5 string bags each. Laboratory analyses were conducted using methods specified by the Polish Committee for Standardization (www.pkn.pl). The content of dry matter (DM)^67^, crude ash (CA)^68^, and crude protein (CP)^69^ was assessed. Also, crude fiber (CF)^70^, crude fat (EE)^71^, starch^72^, NDF^73^, ADF, and ADL^74^ were analyzed. The samples were analyzed in 10 repetitions. The compliance of the content of nutrients declared by the manufacturer was verified, and minor deviations were considered acceptable (Table 5).

**Table 5:** Analytical composition of starter and grower feed for broiler ducks.

Nutrient^c^ (g/kg DM)	Group		SEM
	Control	Zeolite (1%)	
	Starter diet (days 1–28)		
GE (MJ/kg)	16.54	16.41	0.019
DM (g/kg feed)	879.71	878.33	0.345
CA	57.85	65.93	1.032
CP	234.48	243.95	5.637
EE	35.55	35.13	0.270
CF	48.52	52.11	1.783
ADF	62.99	55.39	1.520
NDF	157.53	163.55	3.371
ADL	33.69	27.28	1.303
Starch	437.60	427.70	1.705
Grower diet (days 29–49)			
GE (MJ/kg)	16.43	16.25	0.027
DM (g/kg feed)	889.38	884.94	0.671
CA	59.64	71.20	1.436
CP	193.99	193.99	0.597
EE	40.42	38.51	0.347
CF	56.58	55.23	0.285
ADF	62.94	55.26	2.997
NDF	168.73	171.43	2.051
ADL	25.74	22.82	0.797
Starch	445.54	440.10	1.436

^a,b^Mean values with various letters in the row differ statistically significantly between control and zeolite diets (P < 0.05); each result is done based on 10 repetitions.

^c^*GE* gross energy, *DM* dry matter, *CA* crude ash, *CP* crude protein, *EE* ether extract (crude fat), *CF* crude fiber.

### Growth performance

Ducks were weighed on days 1, 28, and 42 (Radwag, Radom, Poland). The body weight (BW) and feed intake (FI) were monitored. Based on these data, body weight gain (BWG), average daily body weight gain (ADBWG), and average daily feed intake (ADFI) were calculated. The feed conversion ratio (FCR) was calculated based on BW and FI. The parameters were determined for each feeding period and the entire rearing. Moreover, deaths were recorded during rearing (% viability), and production efficiency indicators were calculated, such as the European Production Efficiency Factor (EPEF) and European Broiler Index (EBI). For calculations, formulas were used:

- $$BWG=final \, body \, weight \, \left(g\right)-initial \, body \, weight (g)$$
- $$ADBWG=\frac{BWG\, in \, feeding \, period (g)}{number \, of \, days}$$
- $$ADFI=\frac{total \, feed \, intake \, per \, one \, duck (g)}{42 \, days}$$
- $$FCR=\frac{feed\, intake \, \left(g\right)}{body \, weight \, gain \, \left(g\right)}$$
- $$EPEF=\frac{viability\, \left({\%}\right) \times BW \, (kg)}{\begin{array}{c}age\, \left(days\right) \times FCR \, \left(\frac{kg \, feed}{kg \, gain}\right)\end{array}}\times 100{\%}$$,
- $$EBI = \frac{{\begin{array}{*{20}c} {viability{\mkern 1mu} \left( \% \right) \times ADG\left( {\frac{{\frac{g}{{chick}}}}{{day}}} \right)} \\ \end{array} }}{{FCR\left( {\frac{{kg{\mkern 1mu} feed}}{{kg{\mkern 1mu} gain}}} \right) \times 10}} \times 100\%$$,

### Samples collection

On day 42, 10 birds per group (2 ducks or drakes per pen) with a body weight similar to the average body weight of birds in each pen were selected for slaughter. After previously stunning the birds (with an electric current), they were slaughtered by cutting the spinal cord between the first cervical vertebra and the occipital condyle. After bleeding, the carcasses were dipped in water at 65 °C for 10 s and plucked with a mechanical plucker. Feather leftovers were removed using food-grade wax (Polwax, Jasło, Poland), and the carcasses were eviscerated. The feet were cut off at the ankle joint. Heart, liver, and gizzard were also collected. The prepared carcasses with offal were chilled in a refrigerator (Hendi, Poznań, Poland) at 4 °C for 24 h for further meat quality analyses.

From the digestive tract, approximately a 10 cm long section of the jejunum (from Meckel's diverticulum). The jejunum samples were frozen (Gorenje, Velenje, Slovenia) at − 18 °C.

### Carcass features

Carcasses and offal were weighed. The carcasses were dissected^75^ and weighed. Carcass elements included the neck, pectoral muscles (m. *pectoralis major and* minor), leg muscles (deboned), skin with subcutaneous fat, wings with skin, abdominal fat, and carcass remains (trunk, tibia, and femur). The femur and tibia bones from the right leg were collected and frozen (Gorenje, Velenje, Slovenia) at − 18 °C for further analysis. Based on the results obtained, slaughter yield with or without offal and the percentage of individual carcass elements were calculated.

### Meat quality

During the weighing of the post-chilled carcasses, the pH of the pectoral muscle was measured using a pH meter (Elmetron, Zabrze, Poland) with a dagger electrode. The device was calibrated in standard pH 4.00, 7.00, and 9.00 buffers.

The color of the pectoral and leg muscles was measured with a CR-400 colorimeter (Konica Minolta, Tokyo, Japan) from the inner side of the muscle. The following parameters were determined on the CIE Lab scale: lightness (L*), redness (a*), and yellowness (b*).

The pectoral muscle (previously weighed) was placed in a string bag (notched at the bottom) and then placed in a larger bag to allow water to leak out. It was transferred to a cold store (Hendi, Poznań, Poland) to a temperature of 4 °C for 24 h. After this time, it was re-weighed, and the drip loss was calculated^76^.

Each group's pectoral and leg muscles were ground in a meat grinder (Hendi, Poznań, Poland). Meat samples weighing 0.300 g (± 0.005 g) were weighed. The samples were placed between two pieces of Whatman 1 paper and kept under a load of 2 kg for 5 min. The meat samples were re-weighed, and the water holding capacity (WHC) was calculated^77^.

The chemical composition of pectoral and leg muscles (protein, collagen, salt, intramuscular fat (IMF), and water content) was analyzed, and 90 g of ground meat from each group was weighed. The analyses were performed using the FoodScan apparatus (FOSS, Hillerød, Denmark) using spectrophotometry using near-infrared transmission (NIT)^78^.

Each qualitative analysis of physiochemical features was performed in 10 replicates. For calculations, formulas were used:

- Dressing percentage $$=\frac{carcass\, weight (g)}{live\, body\, weight \left(g\right) }\times 100$$
- Drip loss $=100-\left(\frac{M2}{M1}\right)\times 100\%$ where M2—breast muscle weight after 24 h, M1—initial breast muscle weight.
- $WHC=100-\left(\frac{M2}{M1}\right)\times 100\%$ where, M2—ample weight after 5 min, M1—initial sample weight.

### Jejunum tensile strength and bones’ breaking strength

The jejunum samples were thawed at 4 °C for 24 h. Tensile strength was analyzed using an Instron 3345 device (Instron, Buckinghamshire, England) with Bluehill 3 software. It was defined as the highest force necessary to rupture a sample. Each end of the intestinal sample was placed in pneumatic holders and stretched at a speed of 500 mm/min (Meckel's diverticulum was taken as the standardization point).

The bones were thawed in the same conditions as in the case of the jejunum. Then, they were thoroughly cleaned of meat residues and weighed. Femoral and tibial strength measurements were performed by the Instron Bend Fixture 10 mm Anvil adapter. Each bone was placed on an attachment, and measurements were taken at 250 mm/min speed. The maximum force to fracture a bone and the force per 1 g of bone (N/g) were investigated^25^.

### Statistical analyzes

The numerical data were calculated using a statistical program (Statistica, ver. 13.3.0., TIBCO, Software, Kraków, Poland, 2017). Mean values and SEM were calculated. A one-way analysis of variance for zeolite and sex and a two-way analysis of variance were used, considering the interaction of zeolite and sex. Statistical models were used: a one-way analysis of variance (Y_z/s_ = µ + C_z_/D_s_ + e_z/s_, where Y_z/s_—the dependent variable; µ—the overall mean, C_z_—zeolite in feed, D_s_—sex, e—residual error) for each experimental factor (zeolite addition or sex) and a two-way analysis of variance (Y_z/s_ = µ + C_z_ + D_s_ + CD_zs_ + e_z/s_, where Y_z/s_—the dependent variable; µ—the overall mean, C_z_—zeolite in feed, D_s_—sex, CD_zs_—interaction between sex and zeolite in feed, e—residual error) for interaction between zeolite addition and sex of birds. Statistically significant differences were verified with the Tukey test with a probability of P < 0.05.

### Ethical approval

The consent for research was obtained from the Institutional Animal Care and Use Committee of the Bydgoszcz University of Science and Technology (Local Ethical Committee, No. 2/2022). The ethics committee of Bydgoszcz University of Science and Technology approved the experimental protocols presented in this study. The National Ethical Committee for Animal Experiments recommendations and the ARRIVE guidelines were also considered. The experiment followed Directive 2010/63/EU of the European Parliament and the Council. All methods used in the experiment were applied in accordance with applicable guidelines and requirements.

## Data Availability

None of the data was deposited in an official repository. All of the data obtained in the research are presented in this paper. The data that support the study findings are available from the corresponding author upon reasonable request.
